# Lessons from a systematic literature review of the effectiveness of recombinant factor VIIa in acquired haemophilia

**DOI:** 10.1007/s00277-018-3372-z

**Published:** 2018-05-26

**Authors:** Andreas Tiede, Andrew Worster

**Affiliations:** 1 Hannover Medical School, Department of Haematology, Haemostasis, Oncology and Stem Cell Transplantation, Hannover, Germany; 2 Division of Emergency Medicine, McMaster University, Hamilton, Ontario Canada

**Keywords:** Acquired haemophilia, Bleeding, rFVIIa, Systematic review, Effectiveness, Safety

## Abstract

To conduct a systematic review of the literature reporting efficacy and safety of recombinant factor VIIa (rFVIIa) for the treatment of bleeding in acquired haemophilia and, if data permitted, undertake a meta-analysis of the current evidence. MEDLINE^®^, Embase^®^, and the Cochrane Central Register of Controlled Trials (CENTRAL) databases were searched for all studies on rFVIIa treatment in acquired haemophilia. Heterogeneity of included studies was measured using the inconsistency index (*I*^2^). Of the 2353 publications screened, 290 potentially relevant references were identified: 12 studies published in 32 publications met inclusion criteria. In total, 1244 patients and 1714 bleeds were included (671 patients received rFVIIa treatment for 1063 bleeds). In seven of 12 studies, the initial dose of Recombinant FVIIa was 90 ± 10 μg/kg. Recombinant FVIIa was used as first-line therapy in the majority of cases. Median number of doses administered ranged from 10 to 28. Between 68 and 74% of bleeds were spontaneous, whereas 4–50% were traumatic. Thirty-nine to 90% of bleeds were severe. Haemostatic effectiveness was > 90% in 5/6 studies for both patient and bleed level. Recombinant FVIIa had a favourable safety profile with low risk of general adverse events and thromboembolic-associated events. The heterogeneity of the studies and data precluded a meta-analysis. Recombinant FVIIa demonstrated effectiveness for the treatment of bleeds and had a good safety profile. It is apparent from these data that there is a need for more standardised measures of clinical effectiveness in acquired haemophilia to enable comparison and pooling of results in the future.

**Electronic supplementary material:**

The online version of this article (10.1007/s00277-018-3372-z) contains supplementary material, which is available to authorized users.

## Introduction

Acquired haemophilia A is a rare bleeding disorder that affects approximately 1.48 people per million per year [1]. The incidence of acquired haemophilia increases with age [1], and there is a small peak among women of childbearing age [2, 3]. Acquired haemophilia is caused by autoantibodies against coagulation factor VIII (FVIII) that partially or completely inhibit its procoagulant function [4, 5]. Cases have also been associated with other autoimmune conditions, underlying malignancy, or can be drug-induced [6, 7]. Acquired haemophilia B, caused by antibodies against factor IX, has only been reported in a limited number of patients.

Bleeding presentation in acquired haemophilia can be mild but is more frequently severe (> 67% of cases) [3], and although not all types of bleeding may require intervention (ecchymosis and subcutaneous lesions), the immediate treatment priority is generally to control acute bleeding with bypassing agents [6, 7]. Haemostatic treatment is recommended in patients with acquired haemophilia and active severe bleeding, irrespective of inhibitor titre and residual FVIII activity [8]. Recombinant factor VIIa (rFVIIa) was first approved in 1996 and is indicated for both the treatment of bleeds and prevention of bleeding in patients with acquired haemophilia undergoing surgery or invasive procedures [9].

As acquired haemophilia is a rare disorder, it is difficult to perform fully powered randomised controlled trials. Therefore, the majority of trials supporting the efficacy and safety of rFVIIa in acquired haemophilia have limited numbers of participants, and many are observational and single-arm. The objective of this study, therefore, was to conduct a systematic review of relevant literature and, if data permitted, a meta-analysis of the current evidence for the use of rFVIIa to treat bleeding in acquired haemophilia.

## Materials and methods

### Protocol and registration

#### Study eligibility

Implementation and reporting of the clinical systematic review followed the PRISMA guidelines [10]. Study eligibility criteria followed the Population, Intervention, Comparators, and Outcomes (PICO) framework:

- Population: patients with acquired haemophilia of any age, gender, or race
- Intervention: rFVIIa for the treatment of bleeding in acquired haemophilia
- Comparators: no restriction was placed on the comparator
- Outcomes: haemostatic effectiveness and safety

Included studies met the following pre-defined eligibility criteria: (1) inclusion of patients of any age, gender, or race receiving rFVIIa for the treatment of bleeding in acquired haemophilia (i.e. not patients receiving rFVIIa as prophylaxis to prevent bleeding); (2) randomised, quasi-randomised and non-randomised controlled trial (nRCT) or cohort study; (3) published in English.

The following publication types were excluded from the analysis: (1) reviews and editorials/commentaries (unless they provided any additional data for rFVIIa), (2) case reports/case series, (3) non-human studies, (4) studies with a patient population other than acquired haemophilia, (5) studies investigating a therapy other than rFVIIa, (6) studies enrolling a mixed population, such as congenital haemophilia with inhibitors and acquired haemophilia, but not reporting separate data for acquired haemophilia patients, (7) full-text publications in which there were no data on outcomes of interest with rFVIIa, (8) conference abstracts with no data for rFVIIa dosing or effectiveness, and (9) studies that included less than 10 bleeds of interest. This final criterion was agreed on in advance of the screening of the literature as studies of this size were considered to provide limited, robust data and because of the potential for selection and publication bias. Nine studies were excluded based on this criterion.

### Information sources

The following literature databases were searched on 11 January 2016: MEDLINE^®^ (including MEDLINE^®^ In-Process), Embase^®^, and Cochrane Central Register of Controlled Trials (CENTRAL). No restriction was imposed on the publication timeframe and language in the searches. Bibliographies of identified studies, systematic reviews, and meta-analyses identified through database searches were further utilised for the identification of key studies. Additionally, external experts (the authors) and Novo Nordisk provided feedback on any additional studies or unpublished studies further to those identified by the searches (including non-English publications). This ensured that comprehensive evidence was included in the review. The information was also supplemented by a clinical study report supplied by Novo Nordisk and data available up to the time of the systematic review cut-off date supplied by one of the authors (A Tiede).

### Search strategy, study selection, and data extraction strategy

The electronic search strategy is listed in the Online Resource, Supplementary Table 1. The methodology used was based on National Health Service Centre for Reviews and Dissemination (NHS CRD) [11] and the Cochrane Handbook for Systematic Reviews of Interventions [12]. Results are reported in line with Preferred Reporting Items for Systematic Reviews and Meta-Analyses (PRISMA) guidelines. Comprehensive searches were conducted in a period from database inception to January 2016 to identify studies that were potentially relevant to the project.

Citations retrieved through the literature search were initially screened for inclusion based on their title and abstract. Citations that did not fulfil the inclusion criteria (see ‘Study eligibility’) were excluded, while citations for which eligibility was unclear were retained for further consideration. Following the receipt of all full-text papers, the pre-defined eligibility criteria were applied to the full publications. Screening was performed by two independent reviewers, and any discrepancies between reviewers were reconciled by a third independent reviewer. Through the screening process, various studies were excluded from the feasibility assessment; details on these exclusions are provided in Table 1.

**Table 1:** Number of studies excluded at the second-pass screening (full citations)

Exclusion reason	Number of studies
Review	16
Animal/in vitro	7
Disease	205
Study design	6
Intervention	8
Publications with no rFVIIa effectiveness data^a^	9
No SGA for AH	8
Non-English	1
No extractable data	5
Limited data (fewer than 10 patients)	9
Exclusion reason	Explanation
Review/editorial	If a publication is a review or an editorial/commentary, it was excluded using the ‘Review/editorial’ exclusion criterion
Animal/in vitro	Non-clinical studies, e.g. studies in animals or in vitro systems, were excluded using the ‘Animal/In vitro’ exclusion criterion
Disease	Patients with acquired haemophilia were of interest for this review; if the patient population was different from the population of interest (e.g. CHwI patients), the publication was excluded using the ‘Disease’ exclusion criterion
Intervention	Studies investigating therapy other than rFVIIa were not included in this review and were excluded using the ‘intervention’ exclusion criterion
No sub-group (SGA) for AH	Studies enrolling mixed populations (such as CHwI + AH) but not reporting separate data for AH patients were excluded using the ‘No SGA for AH’ exclusion criterion
No extractable data	Full-text publications in which there were no data pertaining to the haemostatic effectiveness of rFVIIa were excluded using the ‘No extractable data’ exclusion criterion
Language/non-English	Only studies with the full-text publication written in English were included in the review. Studies written in a non-English language were excluded using the ‘Non-English’ exclusion criterion
CA with limited data	Conference abstracts with no data for rFVIIa dosing or effectiveness were excluded using the ‘CA with limited data’ exclusion criterion
Limited data (publications including fewer than 10 eligible patients)	Studies that included fewer than 10 patients of interest (AH treated with rFVIIa) were excluded using the ‘Limited data (publications including fewer than 10 eligible patients)’ exclusion criterion

*AH*, acquired haemophilia; *CA*, conference abstracts; *CHwI*, congenital haemophilia with inhibitors; *rFVIIa*, recombinant factor VIIa; *SGA*, sub-group analysis

^a^Exclusion reason in PRISMA: CA with limited data

Data were extracted from studies that met the eligibility criteria at the second screening in parallel by two independent reviewers. Any discrepancies in the extracted data were reconciled by a third reviewer. Following data extraction, the feasibility of performing analyses to answer the research questions was assessed.

### Definitions and assessment of haemostatic effectiveness

In the analysis on haemostatic effectiveness, it was noted that several scales were used to categorise bleeding control across the studies; effectiveness was judged using two-level (effective, ineffective), three-level ([complete response, partial response, no response] or [effective, partially effective, ineffective]), or four-level scales (excellent, effective, partially effective, ineffective). In line with existing literature in acquired haemophilia, for the purposes of the systematic review, haemostatic effectiveness was defined as complete or partial response.

### Risk of bias in individual studies

The quality of the included studies was assessed using the Downs and Black checklist [13], a validated 26-item checklist for assessing the risk of bias in observational trials and also in single-arm trials and nRCTs [13]. Studies are evaluated for quality of reporting (10 items), external validity (3 items), bias (7 items), and confounding (6 items) using sub-scales of the scoring system (Online Resource, Supplementary Table 2). Quality scores above 20 indicate good quality; 11–20, moderate quality; and below 11, poor quality [14].

### Assessment of feasibility: statistical analysis methods

The performance of a meta-analysis is a two-stage process [15], the first stage involving a calculation of a measure of treatment effect with 95% confidence intervals (CI) for each individual study, followed by, when appropriate, pooling to provide an overall summary statistic.

The data extracted from the studies identified from the systematic review were to be explored according to the proportion of patients whose bleeding was stopped (i.e. ‘patient-level’ data analyses) or proportion of bleeds that were resolved (i.e. ‘bleed-level’ data analyses).

We planned to measure the heterogeneity of included studies using the inconsistency index (*I*^2^) and use a fixed-effect model to pool the results if there was no evidence of measurable heterogeneity or a random-effects model if heterogeneity was present [16] [17].

## Results

### Study selection

**Fig. 1:**
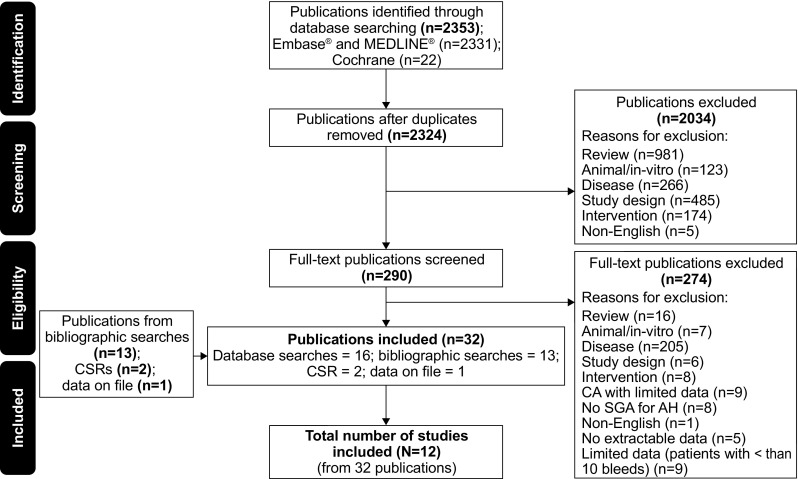
Flow of studies through the systematic review process. AH, acquired haemophilia; CA, conference abstract; CSR, clinical study report; Embase, Excerpta Medica Database; MEDLINE, Medical Literature Analysis and Retrieval System Online; N, number of studies; n, number of publications; SGA, sub-group analysis

A total of 2353 publications published up to (and including) 11 January 2016 were screened. Due to the overlap of coverage between the databases, 29 references found to be duplicates were removed. Following the first-pass review of the citations, 290 potentially relevant references were identified. After a detailed examination of the full-text reports of the relevant studies, 12 studies published in 32 publications (Fig. 1) met the inclusion criteria of the review. Additionally, data for three studies were available from reports provided by Novo Nordisk and an external expert (one a clinical study report for the study conducted by Ma et al. [18], the data for which have subsequently been published [19, 20], the second a report for the study conducted by Seita et al. [21], which has also subsequently been published [22], and the last data on file provided by an author [A Tiede]).

Figure 1 details the flow of studies that were included in the systematic review. A list of excluded studies (*N* = 274) at the second stage of screening, together with reasons for exclusion, is presented in Table 1. Overall, there were 1244 patients included in the 12 studies (671 received rFVIIa for the treatment of bleeds) and 1714 bleeds (1063 treated with rFVIIa) reported. Supplementary Table 3 in the Online Resource lists the included studies following the second-pass review.

### Quality assessment of included studies

Total quality scores for all included studies ranged from 11 to 14, using the Downs and Black checklist, indicating that all studies were of moderate quality (Online Resource, Supplementary Fig. 1) [13, 14]. Scoring of the studies identified that the trial question was specifically stated and well-defined, and the intervention of interest was clearly described in all studies. All studies except one by Borg et al. [2] clearly described the main outcomes. Participant characteristics were clearly described in 10/12 (83.3%) studies. The distribution of principal confounders in each group of patients could not be determined in any of the included studies. With the exception of Luis et al. [23], the main findings were clearly described. Estimates of random variability were described in 4/12 studies (33.3%).

### Study characteristics

The characteristics of the included studies are shown in Supplementary Table 4. Six of the studies provided patient-level data, while six provided data at the bleed level. The majority of included studies were published as journal articles (58.3%); two studies (16.7%) were published as conference abstracts, two were clinical study reports for data subsequently published [18, 22], and one was data on file for GTH (8.3%) provided by the external expert [24]. Two of the studies included > 150 patients [7, 18], while three studies recruited 10 to 30 patients [23, 25, 26]. The number of bleeds was not reported in five of the 12 included studies [2, 23, 25, 27, 28]. Two studies did not report initial dose of rFVIIa [2, 28] and were not included in the subsequently planned meta-analysis. The Zhang et al. study that evaluated a very low dose (25–55 μg/kg) of rFVIIa was also excluded from the subsequently planned meta-analysis [29].

The multiple and obvious differences between studies in terms of populations, interventions, comparators, outcomes, and study designs, and the lack of similarity between sufficient trials precluded any calculation of valid pooled estimate of effectiveness. For these reasons, our results are limited to descriptive summaries.

### Demographic and clinical characteristics

Eleven studies reported data on patient age. The mean age of patients treated with rFVIIa was reported in six studies and ranged from 57.3 to 74 years (Table 2). In three studies, median age was reported and was 59.0, 72.0, and 76.7 years [2, 26, 30], respectively (Table 2). In addition, one study by Scharf et al. [27] reported the age ranges of male (44 to 86 years) and female (20 to 83 years) patients.

**Table 2:** Baseline characteristics reported across included studies

Primary publication	Study design	No. of patients treated	Mean age	Sex (% male)	Mean FVIII level (IU/dL)	Mean inhibitor titre (BU/mL)	Cause of bleeding (%)		Bleeding site (%)								Severity of bleeding (%)		Underlying conditions (%)				Ancillary therapies (%)		Patients with AH treated (%)	
							Traumatic	Spontaneous	Skin	Muscle	Mucosa	Head	Joint	GI tract	Multiple sites	Other	Severe	Non-severe	Autoimmune	Malignancy/cancer	Post-partum	Idiopathic	Anti-fibrinolytic agents	Red blood cells	rFVIIa alone	rFVIIa + other haemostatic agents
Hay 1997 [30]	EAP	38	59^a^	50	–	43^a^	–	–	–	51.3^b^	–	–	9.0^b^	20.5^b^	–	19.2^b^	–	–	–	–	–	–	66^b^	–	–	–
Baudo 2004 [26]	ROB	15	72^a^	60	–	15^a^	4.3^b^	73.9^b^	35^b^	55^b^	–	–	5^b^	–	–	5^b^	95^b^	–	7	13	–	80	6.7	66.7		
Dehmel 2008 [25]	POB	10	76	40	2^a^	10^a^	–	–		10	–	–	–	–	60	30	–	–	–	–	–	–	20	40	–	–
Luis 2010 [23]	ROB	11	–	–	–	–	–	–	–	–	–	–	–	–	–	–	–	–	–	–	–	–	–	–	100	
Dusseldorf study (Scharf 2011a [31])	POB	35	–	68.5	–	–	–	–	62	10.3	–	–	–	13.8	–	13.8	45.7	–	23	8.6	2.9	40	–	–	91.4	8.6
EACH2 registry (Baudo 2012 [7])	Registry	174	73^a^	54.1	2^a^	15.0^a^	24.1	75.9	15.2	59.1	18.8	3.1	3.1	–	0.7	–	89.8	10.1	14.1^**c**^	11.5^**c**^	8.9^**c**^	54.4^c^	17	54.4^**c**^	–	–
HTRS Registry (Ma 2012 [18])^c^	Registry	68	74	49	–	56^d^	21.6^b^	68.4^b^	38.1^b^	20.9^b^	32.3^b^	2.9^b^	15.1^b^	–	–	10.1^b^	–	–	28.3	14.4	3.4	–	–	20.9^b^	64^b^	36^b^
SACHA registry (Borg 2013 [2])^c^	Registry	28	76.7^a^	61	–	16^a^	–	–	61	34	6	1	7	5	–	6	39.3	–	14.63	19.5	7.32	55	–	35.4	28 (93)	2 (7)
Japanese PMS (Seita 2013 [21])^e^	POB	132	67.9^f^	57^f^	4.1^f^	101.1^f^	–	–	12^b^	40^b^	–	4^b^	10^b^	3^b^	–	35^b^	47	–	26.5^f^	12^f^	0^f^	–	–	–	100	–
AHS (Lentz 2014 [28])^c^	Registry	65	66.6	41	–	154.5	–	85	–	–	–	–	–	–	–	–	–	–	34	12	5	–	–	–	–	–
Zhang 2015 [29]^e^	ROB	32	57.3	33.3	5.5	11.3	50	50	25	33.4	25	8.3	–	–	8.3	–	50	50	19.6^c^	12.5^c^	1.8^c^	–	33.3	8.3	12 (37.5)^c^	20 (62.5)^c^
GTH registry (data on file) [24]	Registry	61	71.43	62	3.19	14.6^a^	–	–	29.8^b,c^	42.6^b,c^	–	0.7^b,c^	1.7^b,c^	9.3^b,c^	–	15.9^b,c^	54.3^b^	45.7^b^	20^c^	13^c^	5^c^	67^c^	–	–	–	–

^a^Median value; ^b^Bleed-level data; ^c^Studies in which overall baseline data were captured, as baseline characteristics for patients receiving rFVIIa were not reported separately; ^d^Median highest inhibitor; ^e^Data provided for rFVIIa-only treated group; ^f^rFVIIa monotherapy/combination therapy. Underlying disease and bleed severity in Dusseldorf study is reported for subpopulation of 29 patients in Gheisari 2010 [35]. *AH*, acquired haemophilia; *AHS*, acquired haemophilia surveillance; *EACH2*, European Acquired Haemophilia; *EAP*, extended access program; *FVIII*, factor VIII; *GI*, gastrointestinal; *Hb*, haemoglobin; *HTRS*, Haemostasis and Thrombosis Research Society; *POB*, prospective observational study; *rFVIIa*, recombinant factor VIIa; *ROB*, retrospective observational study; *SACHA*, Surveillance des Auto antiCorps au cours de l’Hémophilie Acquise

Gender was reported for those treated with rFVIIa in 11 of the 12 included studies.

(Table 2). The percentage of males was higher than females in six of the included studies, ranging from 54% [7] to 69% [27], while in four other studies, females were the predominant population (51% [20]; 59% [28]; 60% [25]; 67% [29]). One study by Hay et al. included patients with equal gender distribution [30].

Ethnicity data were reported in just one study by Ma and colleagues [18] and have subsequently been fully published since the time the systematic review was undertaken [20]. Of individuals treated with rFVIIa, Whites/Caucasians made up the largest percentage of the population (73.5%), which included White non-Hispanic (67.6%) and White Hispanic (5.9%) patients. In addition, 23.5% of patients in this study were Black (22.1% were Black non-Hispanic patients and 1.5% were Black Hispanic patients). The remaining 3.0% of patients were of other unspecified races.

Nine studies provided information pertaining to underlying conditions in individuals treated with rFVIIa (Table 2). The most commonly reported underlying conditions were autoimmune or collagen vascular disease (7% [26] to 28% [19]) and malignancy (9% [27] to 20% [2]).

### Recombinant FVIIa treatment regimens

In seven of the 12 studies included in the systematic review, the initial dose of rFVIIa was 90 ± 10 μg/kg (Table 3), while in the study by Dehmel et al., the median initial dose was 105 μg/kg (range 88 to 150 μg/kg) [25]. Across the included studies, the median number of doses administered ranged from 10 reported by Baudo et al. 2004 [26] to 28 reported by Hay et al. 1997 [30]. As there was only a limited number of studies included in each type of analysis (patient level or bleed level), it was not possible to segregate the studies based on mean dose and median dose.

**Table 3:** Treatment regimens of rFVIIa across studies included in the systematic review

Study identifier	Number of patients (Pt) or bleeds (B) treated	Initial dose (μg/kg)			Subsequent doses (μg/kg)	Dosing interval (hours)	Number of doses			Total dose per patient (mg/kg)			Total days of treatment		
		Dose	Min.	Max.			Doses	Min.	Max.	Dose	Min.	Max.	Mean	Min.	Max.
Hay 1997 [30]	Pt = 38	90.4^a^	45	181	–	2^a^	28^a^	1	541	–	–	–	3.9^a^	0.0	43.0
Baudo 2004 [26]^b^	Pt = 8 B = 10	90^a^ (bolus)	46	118	90^a^	2 to 6	10^a^	1	60	–	–	–	2.75^a^	0	8
Dehmel 2008 [25]	Pt = 10	105^a^	88	150	–	2^a^	–	–	–	–	–	–	–	–	–
Luis 2010 [23]	Pt = 11	90	–	–	90^c^	2 to 3	15^c^	1	22	–	–	–	–	–	–
Scharf 2011a [31]	Pt = 35	–	–	–	90–120	2 to 3	–	–	–	–	–	–	–	–	–
Baudo 2012 [7]^d^	Pt = 159 (174 treated first line with rFVIIa)	90^a^	–	–	–	3^a^	12^a^	3	35	84^a^	24	216	–	–	–
Ma 2012 [18]^e^	Pt = 68	90^a^	0.0	–	–	–	14.4^c, f^	1	240	–	–	–	1^a^	–	–
Borg 2013 [2]	Pt = 28	–	–	–	–	–	–	–	–	0.8^c^	0.01	3	4.7^c^	2	33
Seita 2013 [21]^g^	B = 302^g^	99.5^c^	–	–	–	4.6^c^	11.6^c^	–	–	–	–	–	2.9^c^	–	–
Lentz 2014 [28]	Pt = 65	–	–	–	–	–	–	–	–	–	–	–	–	–	–
Zhang 2015 [29]^h^	Pt = 32	40^c^	25	55	–	10^c^	5.5^c^	3	12	22^c^	8	30	–	–	–
GTH Registry (data on file) [24]	B = 51^i^	90^c^	–	–	–	3^a^	–	–	–	250^c^	5	1403	–	–	–

^a^Median value; ^b^Continuous infusion data were excluded; ^c^Mean value; ^d^Efficacy and safety outcomes were reported for 159 and 174 patients, respectively; ^e^Data reported from CSR; ^f^Number of injections; ^g^Data for monotherapy included (302/372 bleeds) and supporting data taken from Amano (data on file); ^h^Data reported for monotherapy/combination therapy; ^i^Number of bleeds with known treatment dose, interval and outcome. *B*, number of bleeds; *Max.*, maximum; *Min.*, minimum; *Pt*, number of patients; *rFVIIa*, recombinant factor VIIa. Note: Borg 2013, Zhang 2015, and Lentz 2014 were included in the description of study characteristics; mean/median is not reported for Luis 2010 [23] initial dose

### Factor VIII level and inhibitor titre

Six of the 12 included studies reported patients’ FVIII levels as mean or median [2, 7, 21, 24, 25, 29]. Mean FVIII levels reported were 3.19 IU/dL in the GTH registry [24], 4.1 IU/dL in the study by Seita et al. 2013 [21], and 5.5 IU/dL by Zhang et al. 2015 [29], while in the remaining three studies, the median FVIII level was 2 IU/dL [2, 7, 25].

Ten studies reported inhibitor titre as mean or median; mean inhibitor titres across the included studies were 11.3 BU/mL reported by Zhang et al. 2015 [29], 101.1 BU/mL by Seita et al. 2013 [21], and 154.5 BU/mL by Lentz et al. 2014 [28], while median inhibitor titres reported ranged from 10.0 BU/mL in the Dehmel et al. 2008 study [25] to 56 BU/mL in the study by Ma et al. subsequently published in Ma et al. 2016 [20] (Table 2).

### Activated partial thromboplastin time and prothrombin time

There were limited data on prothrombin time in the 12 included studies. One study by Zhang et al. reported a mean activated partial thromboplastin time (aPTT) value of 80.0 s [29], and another by Dehmel et al. reported median value of 64 s [25] in individuals treated with rFVIIa. Additionally, one study by Hay et al. reported a median prothrombin time from 12 s pre-rFVIIa treatment to 8.1 s post-treatment [30].

### Bleeding characteristics

#### Type of bleed

Data pertaining to the type of bleeding were reported at two levels: the patient level and the bleed level. At the patient level, in the study by Zhang et al., 50% of patients treated with rFVIIa had spontaneous bleeding and 50% of treated bleeds were traumatic [29], while in the studies by Baudo et al. [7] and Lentz et al. [28], the majority of patients had spontaneous bleeding (75.9 and 85%, respectively). At the bleed level, most bleeds were spontaneous. One study by Ma et al. 2012 (subsequently published in 2016) reported that 68.4% of bleeds treated with rFVIIa were spontaneous, while 21.6% were traumatic [20]. In addition, in the Baudo et al. study, 73.9% of bleeds treated with rFVIIa were spontaneous and 4.3% were traumatic [26].

#### Severity of bleeds

Data on the severity of bleeding were reported in seven of the 12 studies identified in the systematic review, five of which reported data at the patient level [2, 7, 21, 27, 29], and two reported data at the bleed level [24, 26]. In the study by Zhang et al. [29], severe bleeding was defined as life-, limb-, or organ-threatening bleeding; central nervous system bleeding; bleeding with haemoglobin levels < 8 g/dL or a reduction in haemoglobin of 2 g/dL; or bleeding requiring red blood cell (RBC) transfusion of 2 units in 24 h; all other bleeding episodes were considered to be non-severe. Using these definitions, Zhang et al. reported that 50% of patients presented with severe and 50% with non-severe bleeding [29]. Similarly, two studies reported inclusion of 47 and 45.7% patients with severe bleeding episodes, respectively [22, 27]. In the study reported by Borg et al. [2], fewer patients were reported as presenting with severe bleeding (39.3% of patients in the rFVIIa treatment group). In contrast, in Baudo et al., the majority of patients treated with rFVIIa had severe bleeding (89.8%, [7]). At a bleed level, two studies indicated that 54.3 and 95% of bleeds were severe, respectively [24, 26].

### Ancillary therapies

RBC transfusion and anti-fibrinolytic agents were the major ancillary therapies given to patients across the seven studies that reported this information [2, 7, 18, 25, 26, 29, 30]. In the early study by Hay et al., 66% of rFVIIa-treated bleeds were also treated with anti-fibrinolytic agents [30]. In the study by Baudo et al., 54.4% of patients reported RBC transfusion and 18.9% received anti-fibrinolytic agent [7], while Dehmel et al. reported RBC transfusion in 40% of patients with 20% receiving anti-fibrinolytic agents [25]. Zhang et al. reported that 33.3% of patients received anti-fibrinolytic agents as an ancillary therapy with 8.3% reporting RBC transfusion [29]. Using data from an Italian registry, Baudo et al. reported that 6.7% of patients received anti-fibrinolytic agents and 66.7% received RBC transfusion [26]. In Borg et al. 2013, 35.4% of patients received RBC transfusion [2], while in the study by Ma et al. 2012 [18] that was subsequently published in full in 2016 [20], 20.9% received RBC transfusion.

### Line of therapy

At the patient level, three studies reported the use of rFVIIa as first-line treatment in a total of 230/274 patients (83.9%), while 43/274 patients (15.7%) were treated with rFVIIa as second-line treatment [7, 28, 30]. At the bleed level, two studies [18, 26] reported the use of rFVIIa as first-line treatment in 147/159 (92.4%) bleeding episodes overall, while 12/159 (7.6%) bleeding episodes were treated with rFVIIa as second-line treatment.

### Haemostatic effectiveness

**Fig. 2:**
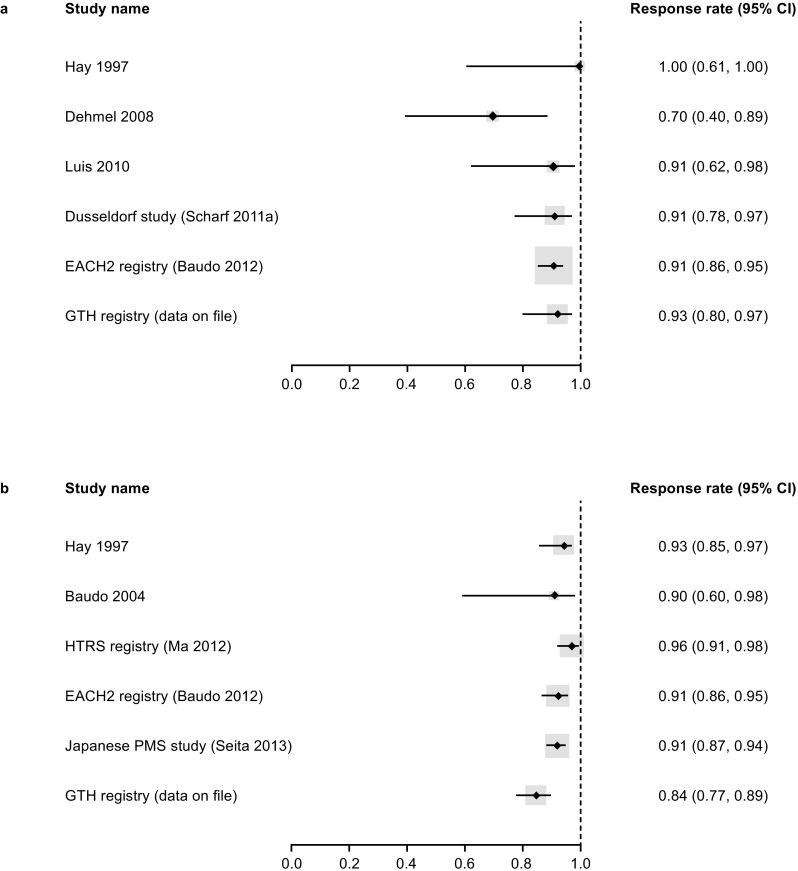
Forest plot for the haemostatic effectiveness using data extracted from the individual studies at **a** patient level (fixed-effect model) and **b** bleed level (random-effects model). CI, confidence interval

There was considerable variability across the 12 included studies in terms of how haemostatic effectiveness was defined (Table 4); as such, when considering if a meta-analysis was feasible, the only effectiveness outcome that provided sufficient data was haemostatic effectiveness, available for six studies, and was defined as complete or partial response. Owing to the small sample size, differences in baseline clinical characteristics between studies, and the differences in how effectiveness was quantified, it was concluded that pooling of the data using a meta-analysis approach was inappropriate. Forest plots showing patient- and bleed-level data extracted for the individual studies were, however, feasible, and are shown in Fig. 2. Haemostatic efficacy was > 90% in five of six studies on the patient level and > 90% in five of six studies on the bleed level.

**Table 4:** Definitions of haemostatic effectiveness reported across the studies

Study identifier	Effectiveness assessor	Assessment of effectiveness	Effectiveness rating scale	Definition of effective/successful treatment
Hay 1997 [30]	Physician	Clinical examination of bleed, careful monitoring of vital signs and full blood count, and ultrasonography or CT scanning where appropriate	Good response, partial response, poor response	Good/partial response is not defined
Baudo 2004 [26]	Physician	Bleed resolution	Very effective, effective, partially effective, ineffective	Very effective is defined as complete cessation of bleeding Effective is defined as residual minor bleeding, and partially effective is defined as reduced but significant bleeding
Dehmel 2008 [25]	Physician	Clinical response (assessed in first 48 h)	Good response, partial response, poor response	A good response is defined as: 1. No dose escalation or switch of bypassing agent 2. Improvement of bleeding-related symptoms 3. Absence of new bleeding sites or symptoms 4. Maximum haemoglobin decrease of 2 g/dL 5. Minimum haemoglobin increase of 1.5 g/dL for every 2 U of RBC Partial response is reported if one of these criteria is not met but bleeding symptoms did not worsen Poor response was reported if more than one of the criteria are not met or if bleeding symptoms worsened
Luis 2010 [23]	NR	Bleed resolution	Complete response	Complete response is not defined
Scharf 2011a [31]	Physician	Bleed resolution	Bleeding controlled, bleeding subsided	Bleeding controlled/subsided is not defined
Baudo 2012 [7]	Physician	Bleed resolution	Resolved, not resolved	Bleed resolution was not defined
Ma 2012 [18]	Physician	Bleed resolution	Bleeding stopped, slowed, no improvement	Bleeding stopped/slowed/no improvement is not defined
Borg 2013 [2]	Physician	Bleed resolution	Complete resolution, improvement in initial bleeding	Complete resolution or improvement in initial bleeding is not defined
Seita 2013 [21]	Physician	Clinical improvement	Markedly effective, effective, moderate, or ineffective	Markedly effective is defined as clinical improvement within 8 h, effective is defined as improvement in 8–12 h, and moderately effective is defined as improvement in > 12 h
Lentz 2014 [28]	NR		Excellent/good, fair/partially effective	Excellent/good/fair/partially effective is not defined
Zhang 2015 [29]	NR	Bleed resolution	Bleeding resolved, bleeding not resolved	Bleeding resolved is defined as bleeding stopped or significantly reduced 1. A stop in bleeding was defined as no need for additional haemostatic medication to achieve initial bleeding control 2. A significant reduction in bleeding was defined as no need for additional haemostatic medication despite noticeable continued bleeding
GTH Registry (data on file) [24]	Physician	Clinical evaluation	Effective, ineffective	A treatment was considered effective if: 1. The clinical condition and symptoms of the bleed did not worsen during the treatment, and 2. No dose increase or change of haemostatic drug was required If these conditions were not met, treatment was considered ineffective

*CT*, computed tomography; *NR*, not reported; *RBC*, red blood cell

### Safety

Recombinant FVIIa had a favourable safety profile with a low risk of general adverse events (AEs) as well as thromboembolic-associated events (Table 5). Ten of the included studies reported information regarding mortality. In eight of these studies, there was no mortality reported as being related to rFVIIa treatment [2, 7, 23, 26, 28–31]. In the study by Seita et al. 2013 [21] that was subsequently published in full [22], four of 132 patients (3.03%) died; two of these patients experienced thromboembolic events while the other two did not. For two of these cases, a causal relationship between rFVIIa therapy and the patients’ deaths was not ruled out by the study authors. In the GTH registry, three out of six deaths were reported that were possibly or probably associated with rFVIIa treatment. One patient died from portal vein thrombosis on day 6 in the study while on rFVIIa for 3 days [24]. The other two patients died of ischemic stroke on the 5th and 35th day of rFVIIa treatment. These two patients received rFVIIa plus tranexamic acid [24].

**Table 5:** Safety of rFVIIa

Study identifier	Intervention	Number of patients treated	Any AE, *n* (%)	Any serious AE, *n* (%)	Any thromboembolic-associated event, *n* (%)	Any cardiovascular-related event, *n* (%)	Any death in patients receiving rFVIIa, *n* (%)	Mortality related to rFVIIa treatment, *n* (%)
Baudo 2004 [26]	rFVIIa	15	–	–	0 (0)	–	4 (26.7)	–
Baudo 2012 [7]	rFVIIa	174	–	–	5 (2.9)	–	29 (16.7)	–
Borg 2013 [2]	rFVIIa	28	–	–	–	–	1 (3.57)	–
Hay 1997 [30]	rFVIIa	38	5 (13.2)	–	1 (2.6)	–	4 (10.5)	–
Lentz 2014 [28]	rFVIIa	65	0 (0)	–	–	–	5 (7.7)	–
Luis 2010 [23]	rFVIIa	11	–	–	0 (0)	–	1 (9.1)	–
Ma 2012 [18]	rFVIIa	68	3 (4.4)	2 (2.9)	1 (1.5)	1 (1.5)	–	–
Scharf 2011a [31]	rFVIIa	35	–	–	–	–	3 (8.6)	–
Seita 2013 [21]	rFVIIa	132	19 (14.4)	6 (4.5)	3 (2.3)	–	4 (3.03)	2 (1.5)^a^
GTH Registry (data on file) [24]	rFVIIa	61	–	–	–	–	6 (9.8)	3 (4.9)^a^
Zhang 2015 [29]	Low-dose rFVIIa (25–55 μg/kg)	12	–	–	0 (0)	–	0 (0)	–
Zhang 2015 [29]	FVIII/low-dose rFVIIa (25–55 μg/kg)	20	–	–	0 (0)	–	0 (0)	–

*AE*, adverse events; *FVIII*, factor VIII; *N*, number of evaluable patients; *n*, number of patients with outcome; *rFVIIa*, recombinant factor VIIa. Relationship of mortality to rFVIIa treatment as reported in the study; ^a^causal relationship between rFVIIa therapy and the patient’s death could not be ruled out

## Discussion

This systematic review includes the largest published collection of data on efficacy and safety outcomes with rFVIIa in acquired haemophilia, including over 1000 individuals who experienced more than 1000 bleeds that were treated with rFVIIa. From these data, it is clear that there is no standard protocol for rFVIIa treatment, and heterogeneity existed in how patients were treated in terms of both the initial dose and the number of doses administered. Similarly, there were differences in the reporting of other patient characteristics of interest, in that factor VIII levels and inhibitor titres were only reported in half the studies, and activated partial thromboplastin time and PTT were reported in only three of the 12 studies included.

The quality of the included studies was judged to be moderate based on the Downs and Black criteria [13], which enabled them to be included in the systematic analysis but limited investigation into the sources of bias. This quality scoring reflects several factors, including that many of the studies were open-label and non-randomised, confounders were not clearly described or adjusted for, and loss to follow up was not clearly described. These limitations reflect some of the difficulties in performing studies in acquired haemophilia, particularly as it is difficult to recruit sizable participant populations given that it is a relatively rare disorder. In addition, many of the studies included in this analysis were observational, partially reflecting the fact that rFVIIa addressed an important medical need in those affected by acquired haemophilia and was widely prescribed for compassionate use. Regardless of the limitations of the studies, and the inability to pool the data to perform a meta-analysis, the results of the systematic review and the data extracted from the individual studies are reliable and informative.

As has been noted previously [32], ideally, in order to enable the pooling of individual studies to produce a single summary estimate, the selected studies should target a common objective and have similar clinical populations and trial design, and accepted reasons for not presenting summary estimates include methodological diversity (different study designs) and clinical diversity (e.g. different metrics and/or outcomes, participant characteristics, or clinical settings) [32]. In this regard, the main issue that limited the pooling of data for the meta-analysis was the heterogeneity in efficacy outcomes, both in terms of the consistency in the definition of bleed responses and the timing of when effectiveness was judged. Of the 12 studies, seven (58%) did not define their measure of effectiveness, and all of them had different terminology for describing responses. Of the five studies that did define haemostatic effectiveness, some defined it in terms of cessation/improvement in bleeding, others included additional criteria in terms of no dose escalation or switch of haemostatic treatment, and one based effectiveness on speed of resolution (markedly effective = improvement in 8–12 h, moderately effective = improvement in > 12 h).

Regardless of this heterogeneity in the definitions employed, haemostatic effectiveness, in terms of the percentage of participants with complete or partial responses or the proportion of bleeds that resolved, was high with responses being, in general, above 90% for both measures in most of the included studies. Most participants experienced spontaneous bleeds, and the most common locations for bleeds included the muscle, skin, the gastrointestinal tract, and mucosa. While the severity of bleeding was heterogeneous with rates of severe bleeding ranging from 40 to 95% between studies, regardless, it is clear that acquired haemophilia has a major impact on the lives of many individuals with the disorder. The safety profile of rFVIIa was positive with very few serious AEs, thromboembolic or cardiovascular events, or deaths associated with rFVIIa.

The issue of inconsistent effectiveness reporting remains relevant in the acquired haemophilia field. For example, a 2015 study of recombinant porcine sequence FVIII assessed efficacy based on bleed control, overall clinical status, and FVIII activity levels [33]. This definition of effectiveness differs from the assessment criteria in other studies of FVIII. Hence, due to the limitation in terms of participant numbers and the lack of a common standard for efficacy assessments, it is difficult to make robust assessments of treatment effectiveness or combine data sets to generate more statistically meaningful findings. Going forward, standardised definitions and outcome measurements are required in acquired haemophilia, as otherwise it is impossible to quantitatively evaluate the risk of adverse events and the benefit of therapeutic interventions. With this in mind, the Definitions in Acquired Haemophilia project is currently being undertaken by the Factor VII, Factor IX, and Rare Coagulation Disorders Subcommittee of the Scientific and Standardization Committee of the International Society on Thrombosis and Haemostasis. This project aims to establish uniform definitions for haemostatic outcomes, the cessation of bleeds, and the remission and relapse of disease. Such standardisation would also enable much more effective interrogation of the evidence base and allow sub-group analysis of predictive factors for better or worse treatment outcomes [34].

## Conclusions

In conclusion, in this comprehensive review of published data for individuals with acquired haemophilia, rFVIIa demonstrated effectiveness for the treatment of bleeds and had a positive safety profile. It is apparent from these data that given the difficulty in performing large randomised studies for rare bleeding disorders, there is a need for more standardised measures of clinical effectiveness in acquired haemophilia to enable data comparison and pooling in the future.

## Electronic supplementary material

ESM 1 (DOCX 67 kb)
