# Supplementary material for: Lessons from a systematic literature review of the effectiveness of recombinant factor VIIa in acquired haemophilia
Source: Ann Hematol. 2018 May 26;97(10):1889–901. doi: 10.1007/s00277-018-3372-z (PMC6208690; doi:10.1007/s00277-018-3372-z)
Supplement: Supplementary file 1 — (DOCX 67 kb) [file 277_2018_3372_MOESM1_ESM.docx]

**Lessons from a systematic literature review of the effectiveness of recombinant factor VIIa in acquired haemophilia**

Journal: Annals of Hematology

Andreas Tiede* and Andrew Worster

*Haematology, Haemostasis, Oncology and Stem Cell Transplantation, Hannover Medical School, Hannover, Germany. E-mail: [tiede.andreas@mh-hannover.de](mailto:tiede.andreas@mh-hannover.de)

**Supplementary Table 1** Search strategies

Embase® and MEDLINE® search strategy (11 January 2016)

| No. | Search terms | Facet | Results |
| --- | --- | --- | --- |
| #1 | 'recombinant blood clotting factor 7a'/exp | Interventions | 5,675 |
| #2 | 'haemophilia'/exp OR 'haemophilia' | Disease | 35,395 |
| #3 | #1 AND #2 | Final combination | 2,316 |
| #4 | #3 AND [animals]/lim NOT ([humans]/lim AND [animals]/lim) | Excluding animal studies | 39 |
| #5 | #3 NOT #4 | Final number | 2,277 |

Cochrane search strategy (11 January 2016)

| No. | Search terms | Facet | Results |
| --- | --- | --- | --- |
| #1 | “eptacog alfa” or “eptacog alfa activated” or “eptacog alfa pegol” or “eptacog alfa pegol activated” or “eptacog beta” or “eptacog beta (activated)” or “human coagulation factor VII (activated)” or “marzeptacog alfa” or “marzeptacog alfa (activated)” or “marzeptacog alpha” or “niastase” or “nn 1731” or “nn1731” or “novo seven” or “novoseven” or “oreptacog alfa” or “oreptacog alfa (activated)” or “oreptacog alpha” or “pegylated recombinant factor VIIa” or “recombinant coagulation factor VIIa” or “recombinant factor VIIa” or “vatreptacog alfa” or “vatreptacog alfa (activated)” or “vatreptacog alpha” | Interventions | 200 |
| #2 | MeSH descriptor: [Hemophilia A] explode all trees | Disease | 200 |
| #3 | #1 AND #2 | Final combination | 35 |
| #4 | # 4 in Cochrane Reviews, Other Reviews, Trials and Methods Studies | Limited to Cochrane review trials and method studies | 22 |

MEDLINE® in-process search strategy using the PubMed® platform (11 January 2016)

| No. | Search terms | Facet | Results |
| --- | --- | --- | --- |
| #1 | “eptacog alfa” or “eptacog alfa activated” or “eptacog alfa pegol” or “eptacog alfa pegol activated” or “eptacog beta” or “eptacog beta (activated)” or “human coagulation factor VII (activated)” or “marzeptacog alfa” or “marzeptacog alfa (activated)” or “marzeptacog alpha” or “niastase” or “nn 1731” or “nn1731” or “novo seven” or “novoseven” or “oreptacog alfa” or “oreptacog alfa (activated)” or “oreptacog alpha” or “pegylated recombinant factor VIIa” or “recombinant coagulation factor VIIa” or “recombinant factor VIIa” or “vatreptacog alfa” or “vatreptacog alfa (activated)” or “vatreptacog alpha” | Interventions | 949949 |
| #2 | Search Hemophilia | Disease | 24857 |
| #3 | #1 AND #2 | Final combination | 1533 |
| #4 | Search #3 AND (pubstatusaheadofprint OR inprocess[sb]) | Limited to ahead of print and in-process citations | 54 |

Total number of citations retrieved from all databases

| Database | Numbers retrieved |
| --- | --- |
| Embase® and MEDLINE® | 2,277 |
| MEDLINE® in-process | 54 |
| CENTRAL | 22 |
| **Total** | **2,353** |

**Supplementary Table 2** Downs and Black quality assessment checklist

| Quality of reporting (10 items) |
| --- |
| 1. Is the hypothesis/objective of the study clearly described? 2. Are the main outcomes to be measured clearly described in the introduction or methods section? 3. Are the characteristics of the patients included in the study clearly described? 4. Are the interventions of interest clearly described? 5. Are the distributions of principal confounders in each group of subjects to be compared clearly described? 6. Are the main findings of the study clearly described? 7. Does the study provide estimates of the random variability in the data for the main outcomes? 8. Have all important adverse events that may be a consequence of the intervention been reported? 9. Have the characteristics of patients lost to follow-up been described? 10. Have actual probability values been reported (e.g., 0.035 rather than <0.05) for the main outcomes except where the probability value is less than 0.001? |
| External validity (3 items) |
| 1. Were the subjects asked to participate in the study representative of the entire population from which they were recruited? 2. Were those subjects who were prepared to participate representative of the entire population from which they were recruited? 3. Were the staff, places, and facilities where the patients were treated representative of the treatment the majority of the patients received? |
| Bias (7 items) |
| 1. Was an attempt made to blind study subjects to the intervention they received? 2. Was an attempt made to blind those measuring the main outcomes of the intervention? 3. If any results of the study were based on "data dredging", was this made clear? 4. In trials and cohort studies, do the analyses adjust for different lengths of follow-up of patients, or in case-control studies, is the time period between the intervention and outcome the same for cases and controls? 5. Were the statistical tests used to assess the main outcomes appropriate? 6. Was compliance with the interventions reliable? 7. Were the main outcomes measures used accurate (valid and reliable)? |
| Confounding (6 items) |
| 1. Were the patients in different intervention groups (trials and cohort studies), or were the cases and controls (case-control studies), recruited from the same population? 2. Were study subjects in different intervention groups (trials and cohort studies), or were the cases and controls (case-control studies), recruited over the same period of time? 3. Were study subjects randomised to intervention groups? 4. Was the randomised intervention assignment concealed from both patients and healthcare staff until recruitment was complete and irrevocable? 5. Was there adequate adjustment for confounding in the analyses from which the main findings were drawn? 6. Were losses of patients to follow-up taken into account? |

**Supplementary Table 3** Studies included in the systematic review following the second-pass review

| Study identifier | Title | Authors | Publication year | Journal name | Volume |
| --- | --- | --- | --- | --- | --- |
| Baudo 2004 [1] | Treatment of acquired factor VIII inhibitor with recombinant activated factor VIIa: data from the Italian registry of acquired haemophilia | Baudo F; De Cataldo F; Gaidano G | 2004 | Haematologica | 89 |
| Baudo 2012 [2] | Management of bleeding in acquired haemophilia A: Results from the European Acquired Haemophilia (EACH2) registry | Baudo F; Collins P; Huth-Kühne A; Lévesque H; Marco P; Nemes L; Pellegrini F; Tengborn L; Knoebl P | 2012 | Blood | 120 |
| Borg  2013 [3] | Outcome of acquired haemophilia in France: The prospective SACHA (Surveillance des Auto antiCorps au cours de l'Hémophilie Acquise) registry | Borg JY; Guillet B; Le Cam-Duchez V; Goudemand J; Levesque H | 2013 | Haemophilia | 19 |
| Dehmel 2008 [4] | Thrombelastographic monitoring of recombinant factor VIIa in acquired haemophilia | Dehmel H; Werwitzke S; Trummer A; Ganser A; Tiede A | 2008 | Haemophilia | 14 |
| Gheisari 2010 [5] | Clinical features and outcome of acquired haemophilia A: Interim analysis of the Dusseldorf study | Gheisari R; Bomke B; Hoffmann T; Scharf RE | 2010 | Hamostaseologie | 30 |
| Hay  1997 [6] | The treatment of bleeding in acquired haemophilia with recombinant factor VIIa: A multicentre study | Hay CR; Negrier C; Ludlam CA | 1997 | Thrombosis and Haemostasis | 78 |
| HTRS (data on file) [7] | Haemostasis and Thrombosis Research Society Registry Update: Treatment of Bleeding in Patients with Acquired Haemophilia | - | - | - | - |
| Lentz 2010 [8] | The acquired haemophilia surveillance (AHS) project: A novel mechanism of capturing post-marketing safety information on RFVIIA (Novoseven^®^RT) in acquired haemophilia | Lentz SR; Tandra A; Peters TJ; Cooper DL | 2010 | Blood | 116 |
| **Lentz 2011** [9] | A novel mechanism of capturing post-marketing safety information on recombinant factor VIIa (rFVIIa) in the rare disorder acquired haemophilia: the acquired haemophilia surveillance (AHS) project | Lentz S; Tandra A; Doucette K; Cooper DL | 2011 | Value in Health | 14 |
| **Lentz 2012** [10] | A novel approach to capturing post-marketing safety information on recombinant factor VIIa (RFVIIA) in acquired haemophilia: Final data from the AHS project | Lentz SR; Tandra A; Gut RZ; Cooper DL | 2012 | Blood | 120 |
| **Lentz 2014** [11] | A novel supplemental approach to capturing post-marketing safety information on recombinant factor VIIa in acquired haemophilia: The Acquired Haemophilia Surveillance project | Lentz SR; Tandra A; Gut RZ; Cooper DL | 2014 | Journal of Blood Medicine | 5 |
| **Luis 2010** [12] | Acquired haemophilia A (AHA): Management profiles in Argentina | Luis A; Alejandro A; Pierdominicci M; Azzaro A; Cermelj M; Bernard H | 2010 | Haemophilia | 16 |

AH, acquired haemophilia; AHS, acquired haemophilia surveillance; HTRS, Haemostasis and Thrombosis Research Society, rFVIIa, recombinant factor VIIa.

**Supplementary Table 4** Characteristics of included studies

| Total sample size | Number of studies | Study identifier | Total number of patients | Number of patients treated with rFVIIa |
| --- | --- | --- | --- | --- |
| 10–30 | 3 | Dehmel 2008; Luis 2010; Baudo 2004  [1,4,12] | 42 | 36 |
| 31–50 | 2 | Scharf 2011a; Hay 1997  [6,13] | 73 | 73 |
| 51–100 | 4 | Lentz 2014; Borg 2013; Zhang 2015; GTH Registry (data on file) [3,14,11,15] | 330 | 188 |
| 101–150 | 1 | Seita 2013 [16] | 132 | 132 |
| >150 | 2 | Ma 2012; Baudo 2012 [2,17] | 667 | 242 |
| Total | 12 |  | 1244 | 671 |

rFVIIa, recombinant factor VIIa.

**Supplementary Figure 1** Total quality scores for included studies


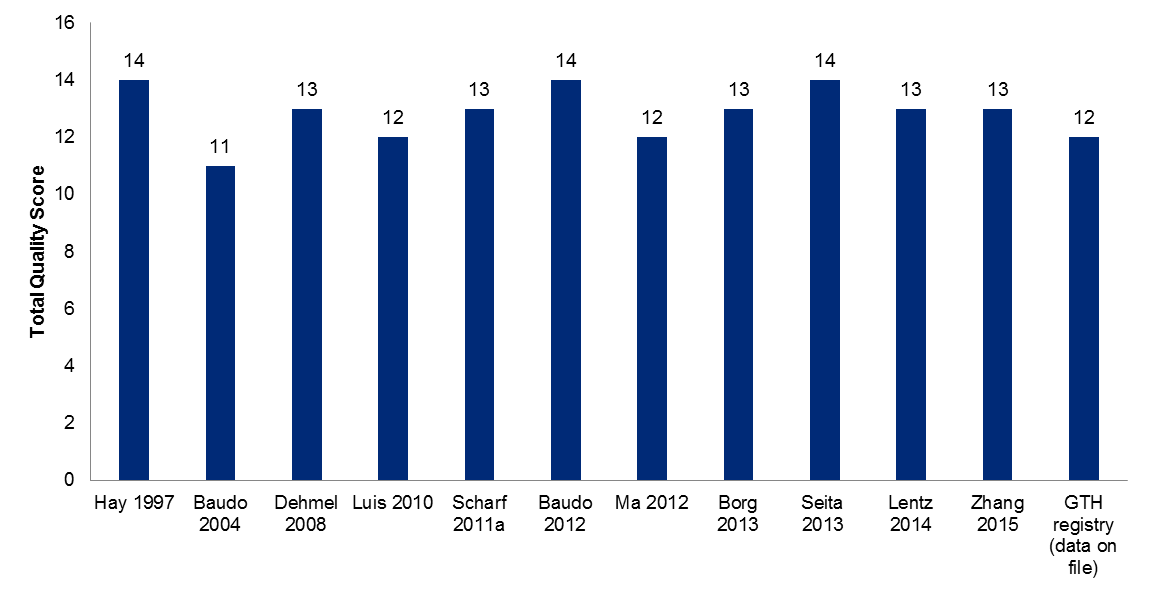


**References**

1. Baudo F, de Cataldo F, Gaidano G (2004) Treatment of acquired factor VIII inihibitor with recombinant activated factor VIIa: data from the Italian registry of acquired hemophilia. Haematologica 89 (6):759–760

2. Baudo F, Collins P, Huth-Kuhne A, Levesque H, Marco P, Nemes L, Pellegrini F, Tengborn L, Knoebl P, contributors Er (2012) Management of bleeding in acquired hemophilia A: results from the European Acquired Haemophilia (EACH2) Registry. Blood 120 (1):39–46. doi:10.1182/blood-2012-02-408930

3. Borg JY, Guillet B, Le Cam-Duchez V, Goudemand J, Levesque H, Group SS (2013) Outcome of acquired haemophilia in France: the prospective SACHA (Surveillance des Auto antiCorps au cours de l'Hemophilie Acquise) registry. Haemophilia 19 (4):564–570. doi:10.1111/hae.12138

4. Dehmel H, Werwitzke S, Trummer A, Ganser A, Tiede A (2008) Thrombelastographic monitoring of recombinant factor VIIa in acquired haemophilia. Haemophilia 14 (4):736–742. doi:10.1111/j.1365-2516.2008.01759.x

5. Gheisari R, Bomke B, Hoffmann T, Scharf RE (2010) Clinical features and outcome of acquired haemophilia A. Interim analysis of the Dusseldorf study. Hamostaseologie 30 (3):156-161

6. Hay CR, Negrier C, Ludlam CA (1997) The treatment of bleeding in acquired haemophilia with recombinant factor VIIa: a multicentre study. Thromb Haemost 78 (6):1463–1467

7. HTRS (data on file) Haemostasis and Thrombosis Research Society Registry Update: Treatment of Bleeding in Patients with Acquired Haemophilia.

8. Lentz SR, Tandra A, Peters TJ, Cooper DL (2010) The acquired haemophilia surveillance (AHS) project: A novel mechanism of capturing post-marketing safety information on RFVIIA (Novoseven®RT) in acquired haemophilia. Blood 116 (21):3674

9. Lentz S, Tandra A, Doucette K, Cooper DL (2011) A novel mechanism of capturing post-marketing safety information on recombinant factor VIIa (rFVIIa) in the rare disorder acquired haemophilia: the acquired haemophilia surveillance (AHS) project. Value in Health 14 (3):A60. doi:10.1016/j.jval.2011.02.340

10. Lentz SR, Tandra A, Gut RZ, Cooper DL (2012) A novel approach to capturing post-marketing safety information on recombinant factor VIIa (RFVIIA) in acquired haemophilia: Final data from the AHS project. Blood 120 (21):3371

11. Lentz SR, Tandra A, Gut RZ, Cooper DL (2014) A novel supplemental approach to capturing post-marketing safety information on recombinant factor VIIa in acquired hemophilia: the Acquired Hemophilia Surveillance project. J Blood Med 5:1–3. doi:10.2147/JBM.S55216

12. Luis A, Alejandro A, Pierdominicci M, Azzaro S, Cermelj M, Bernard H (2010) Acquired haemophilia A (AHA): Management profiles in Argentina. Haemophilia 16 ((Suppl 4))

13. Scharf RE, Gheisari R, Bomke B, Hoffmann T (2011) Improved prognosis of acquired hemophilia A (AHA): Results of the Duesseldorf monocenter study. J Thromb Haemost 9 ((Suppl. 2)):921

14. GTH Registry (data on file) (2016) GTH Registry on Acquired Haemophilia: A Management of Bleeds.

15. Zhang XH, Zhu XL, Niu T, Sun J, Liu H, Feng R, Yang LH, Wei Q, Ma QH, Wang QM, Feng FE, Fu HX, Mo XD, Lv M, Huang XJ (2015) Combination of FVIII and low-dose rFVIIa improves haemostasis in acquired haemophilia A patients: a collaborative controlled study. Thromb Res 135 (5):835–840. doi:10.1016/j.thromres.2015.02.029

16. Seita I, Amano K, Higasa S, Sawada A, Kuwahara M, Shima M (2013) Treatment of acute bleeding episodes in acquired haemophilia with recombinant activated factor VII (rFVIIa): Analysis from 10-year Japanese postmarketing surveillance. J Thromb Haemost 11 ((Suppl. 2)):119

17. Ma AD, Kessler CM, Al-Mondhiry HAB, Fisher M, Gut RZ, Cooper DL (2012) Use of Recombinant Factor VIIa (rFVIIa) for Acute Bleeding Episodes in Acquired Hemophilia: Final Analysis From the Hemostasis and Thrombosis Research Society (HTRS) Registry AH Study. Blood 120 (21):4624
